# Sociodemographic and Clinical Determinants on Health-Related Quality of Life in Emerging Andalusian Adults with Type 1 Diabetes: A Cross-Sectional Study

**DOI:** 10.3390/jcm13010240

**Published:** 2023-12-31

**Authors:** María-Ángeles Núñez-Baila, Anjhara Gómez-Aragón, José Rafael González-López

**Affiliations:** Nursing Department, Faculty of Nursing, Physiotherapy and Podiatry, Universidad de Sevilla, 41009 Seville, Spain; mnbaila@us.es (M.-Á.N.-B.); joserafael@us.es (J.R.G.-L.)

**Keywords:** emerging adulthood, type 1 diabetes, Health-Related Quality of Life, cross-sectional study

## Abstract

(1) Background: Having type 1 diabetes during emerging adulthood can impact quality of life due to the challenge of balancing optimal glycemic blood levels with a period of transition and exploration. The purpose of this study was to characterize the quality of life of emerging adults aged 18 to 29 years with type 1 diabetes and to determine the associations between dimensions of Health-Related Quality of Life in type 1 diabetes and sociodemographic and diabetes-related variables. (2) Methods: This cross-sectional descriptive study was conducted in Andalusia, Spain, from October 2021 to July 2022. A total of 362 emerging adults with type 1 diabetes (67.4% women, mean age 22.8 ± 3.4 years) participated. Data were gathered via sociodemographic information form and the ViDa1 scale. Statistical evaluations, encompassing descriptive analyses, *t*-tests, ANOVA, Pearson correlations, and logistic regression, were conducted using SPSSv26, adhering to STROBE guidelines. (3) Results: Among the participants, 52.1% have a glycosylated hemoglobin level over 7%. Interference with Life is correlated with sex, age, and age at diagnosis, with age being the only predictor. Self-Care is correlated with and predicted by glycosylated hemoglobin levels. Well-being is correlated with and predicted by sex, Body Mass Index, and glycosylated hemoglobin levels. Concern about the Condition is correlated with and predicted by sex and glycosylated hemoglobin levels. (4) Conclusions: Despite concerns about their disease, participants generally maintain optimal levels of Health-Related Quality of Life in type 1 diabetes. Predictive factors for Health-Related Quality of Life in type 1 diabetes in this group include sex, age, Body Mass Index, and glycosylated hemoglobin.

## 1. Introduction

Type 1 diabetes mellitus (T1DM) is predominantly diagnosed during childhood and persists as a chronic condition into adulthood [1]. The phase of emerging adulthood, spanning ages 18 to 29 years, presents unique challenges. This period is characterized by significant life transitions, including extended education, fluctuating job markets, evolving life expectations, and shifting societal roles [2,3].

This transitionary phase is also characterized by an identity exploration of values in the social, educational, and personal spheres, striving for self-improvement and goal attainment. While emerging adults generally enjoy good health and no concerns regarding their well-being [4], those living with T1DM may experience a substantial impact on their Health-Related Quality of Life (HRQoL) [5,6].

From childhood to emerging adulthood, blood glucose levels decrease, with the latter phase being a critical period [1,7,8]. Only 17% of early emerging adult (ages 18–25) and 30% of later emerging adults (ages 26–30) met the American Diabetes Association recommendations for glycemic target [9]. Comparable percentages are evident in the Global TEENs Study [10], where merely 18.4% of emerging adults reached the glycosylated hemoglobin A1C (A1C) target. Similarly, in the study by Carels et al. [11], only 18% of emerging adults with T1DM showed an A1C level below 53 mmol/mol. This is compounded by the finding that HRQoL in T1DM is intricately linked to glycemic blood levels, with higher A1C values correlating with lower HRQoL [10,12,13]. The complexity of this relationship is accentuated in emerging adults when compared to those with type 2 diabetes [14,15]. The longitudinal study conducted by Stahl-Pehe et al. [16] indicated a potential inverse correlation between a decline in HRQoL for individuals with T1DM and a reduction in A1C levels over a three-year period, though it did not conclusively establish causality.

The HRQoL in people with T1DM is influenced by a myriad of factors. These encompass disease duration [1], sex disparities with women often reporting lower HRQoL [6,13,15,17], and the post-high school transition to independence [18]. A diagnosis during emerging adulthood [19], especially in the latter stage (25–29 years), is associated with heightened anxiety and a subsequent decline in HRQoL in this demographic [20]. Moreover, lifestyle practices such as consistent exercise, diligent daily monitoring of blood glucose, carbohydrate counting, and tailored insulin adjustment have been linked to improved HRQoL in emerging adults with T1DM [10].

In addition to life transitions characteristic of emerging adulthood, including university enrollment and evolving social relationships [21], there exists a significant gap in the literature concerning HRQoL studies for this particular age group with T1DM [6,10,14,20]. This is in stark contrast to the extensive research focused on children and adolescents [7,22,23,24,25]. Notably, emerging adulthood, a phase often marked by experimentation and risky behaviors, reveals no substantial differences in activities like alcohol consumption, tobacco use, or physical inactivity between individuals with T1DM and their peers without T1DM [3,26].

While there is a vast knowledge regarding the transition from pediatric to adult care for individuals with T1DM, the following life stage has comparatively garnered less focus [27]. In the treatment of chronic conditions like T1DM, optimizing HRQoL is an end in itself. This study seeks to bridge this gap by characterizing the HRQoL among emerging adults with T1DM in Andalusia and exploring the association between HRQoL dimensions (including Interference with Life, Self-Care, Well-Being, and Concern about the Condition) and various sociodemographic and clinical factors. We hypothesize that a favorable HRQoL in T1DM among emerging adults is influenced by factors such as blood glucose levels (A1C), duration of diabetes, timing of diagnosis during emerging adulthood, and life challenges and independence milestones like employment status or living arrangements. 

## 2. Materials and Methods

### 2.1. Study Design and Population

This descriptive, cross-sectional study was conducted in Andalusia, Spain, from October 2021 to July 2022. It included both women and men aged between 18 and 29 years, diagnosed with T1DM for at least one year, and residing in Andalusia. According to the 2021 Andalusian database, there were 5991 individuals in this age group diagnosed with T1DM. Using a 95% confidence interval, a representative sample of 361 emerging adults with T1DM was assigned for the study.

**Figure 1:**
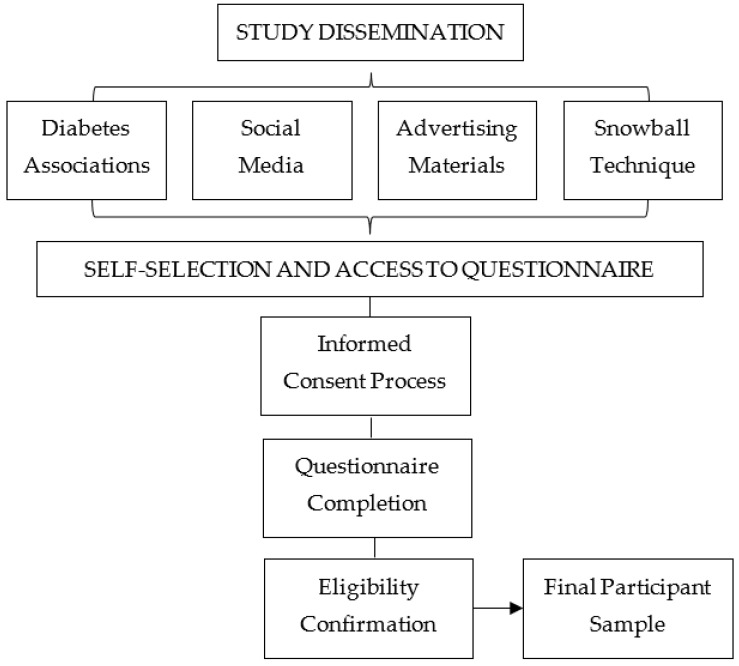
Flow chart of participant enrollment process.

The sample was purposively recruited through different diabetes associations in Andalusia and complemented by outreach social media and advertising materials. The process of participant enrollment is detailed in Figure 1. The voluntary nature of participation could introduce self-selection bias, as those who chose to participate might have motivations unrelated to T1DM. Participants were asked to complete an online questionnaire via Google Forms, preceded by an explanatory note with the researcher’s contact information. Written informed consent was secured from all participants before they began the questionnaire.

### 2.2. Measures

The questionnaire was designed to collect sociodemographic data, including sex, date of birth, age, weight, height, civil status, province of residence in Andalusia, living situation, and current study and employment statuses. Additionally, it gathered health-related information, focusing on variables pertinent to T1DM, such as coexisting conditions, the time of T1DM diagnosis, and A1C values. The questionnaire also assessed HRQoL in individuals with T1DM.

The HRQoL in T1DM variable was measured using the ViDa1 questionnaire (Likert type, 1–5) with 34 items grouped in 4 dimensions [28]. The score ranges for these dimensions are as follows: Interference with Life dimension from 12 to 60 (Cronbach’s α = 0.86), Self-Care from 10 to 50 points (Cronbach’s α = 0.84), Well-Being from 5 to 25 points (Cronbach’s α = 0.76), and Concern about the Condition from 4 to 20 points (Cronbach’s α = 0.71). Due to the subjective nature of this tool, it does not have cut-off points. In this context, higher scores in Interference with Life and Concern about the Condition indicated lower HRQoL in T1DM, whereas higher scores in Self-Care and Well-Being, reflect improved HRQoL in T1DM. After a consultation with the corresponding author of the article about the tool [28], HRQoL in T1DM was categorized as high or low based on mean scores obtained in each dimension.

### 2.3. Ethical Considerations

The study was conducted in accordance with the principles of the Declaration of Helsinki. It received ethical approval by the Ethics Committee of the University Hospitals Virgen Macarena and Virgen del Rocío, under the code 2150-M1-22, as documented in their proceedings dated 11 February 2020. Participants were informed about the voluntary and anonymous nature of their participation, with assurance that their data would be exclusively utilized for scientific purposes. Prior to completing the questionnaire, written consent for the processing of anonymized data was obtained. To maintain privacy, the questionnaire did not request any personal information, and location data were analyzed at the provincial level.

### 2.4. Data Analysis

Descriptive statistical measures, including frequency distributions, means, and standard deviations were employed to illustrate participant sociodemographic characteristics of participants and the scores from the ViDa1 questionnaire.

Unpaired T-tests for independent samples were utilized to evaluated differences in HRQoL in T1DM based on sex, age, A1C, age at T1DM diagnosis, presence of coexisting conditions, and current educational enrollment. To compare HRQoL in T1DM dimensions across different civil and employment statuses, regions in Andalusia, Body Mass Index (BMI), living situations, and educational levels, ANOVA was utilized, supplemented with the Bonferroni test where necessary. The Kolmogorov–Smirnov and Levene’s tests were applied to verify the normality and the homoscedasticity of each variable, respectively. The Kolmogorov–Smirnov test indicated a non-normal distribution of the sample, while Levene’s test evaluated homoscedasticity, noting exceptions in variance equality in the Well-Being (Civil status), Interference with Life (Andalusian province), and Self-Care (Level of studies) dimensions.

Despite potential normality violations, the robustness of the sample size allowed for the application of parametric Student’s t-test and ANOVA. This approach is justified by the absence of extreme bias or flat distributions and a sufficiently large sample size (*n* > 30), conforming to the central limit theorem [29]. Cohen’s d coefficient and eta square (η²) were utilized to estimate the effect size, with thresholds set at 0.2 and 0.01 for a “small” effect size, 0.5 and 0.06 for a “medium” effect size, and 0.8 and 0.14 for a “large” effect size, respectively [30].

Pearson correlation analyses were applied to assess associations among sex (treated as a dummy variable), age, BMI, A1C levels, age at diagnosis, and duration of diabetes. Regression analyses for each HRQoL dimension in T1DM were conducted, with the Goodness of Fit evaluated through homoscedasticity and independence assessment in the residuals, using scatter plots and the Durbin–Watson statistic. The Grubb’s Outlier Test was applied to identify discrepancies between observed and expected values. The variables demonstrated both normality and a linear correlation. Moreover, multicollinearity was ruled out, as evidenced by Factor Inflation Variance and Tolerance values. Four distinct multiple regression models are presented, corresponding to each dimension of the ViDa1 questionnaire, which served as the dependent variables. Independent variables were selected based on significant Pearson correlation coefficients with the ViDa1 dimensions. Additionally, no corrections for multiple testing were applied, which heightens the risk of type I errors. For instance, a Bonferroni correction would have adjusted the significance level for the four conducted regression models, thereby reducing the likelihood of false positives [31]. All analyses were performed using SPSS version 26.0, with a *p*-value less than 0.05 deemed statistically significant. Lastly, the research adhered to Strengthening the Reporting of Observational Studies in Epidemiology (STROBE) guidelines, ensuring the reproducibility and result presentation quality. 

## 3. Results

### 3.1. Characteristics of the Sample

Out of 525 initial respondents to the questionnaire, 163 were excluded for not meeting the inclusion criteria, resulting in a final sample of 362 emerging adults with T1DM. The average age of the participants was 22.8 years (SD 3.4), with women constituting 67.4% of the sample. The average age of T1DM diagnosis was 10.9 years (SD 6.7, range: 0–28), and the mean duration since diagnosis was 11.9 years (SD 6.6) (range: 1–28). In addition, over half of the participants had an A1C level above 53 mmol/mol (Table 1).

Overall, emerging adults with diabetes generally reported a favorable HRQoL in the context of T1DM. More than half declared a low Interference with Life (79.6%), along with high levels of Self-Care (76.5%) and Well-Being (64.4%). However, a notable 68.5% of participants expressed considerable concern about their condition, with hyperglycemia and the potential for future diabetes complications being the most concerning factors. Detailed item-wise and overall scores for each dimension of HRQoL are presented in Table S1.

The most prevalent coexisting conditions among this sample included food allergies (20.4%), skin problems (16.9%), and various other conditions (10.5%). Notably, hypothyroidism (9.9%), asthma (8.3%), celiac disease (7.5%), obesity (6.4%), gastroparesis (5.5%), lactose intolerance (4.7%), food allergy (4.4%), cardiovascular disease (2.8%), and mental health disorder (1.4%) were also observed. Table S2 provides a comprehensive overview of how these health conditions influenced each HRQoL dimension in T1DM.

### 3.2. Relationships of Participants’ Quality of Life with Their Demographic Characteristics and Clinical Diabetes Determinants

For each HRQoL dimension in the context of T1DM, mean scores were compared across independent samples using the unpaired Student’s t-test. This statistical approach was utilized to identify potential differences in HRQoL outcomes based on several key variables: sex, age group, A1C levels, timing of the diagnosis, current student status, and the presence of coexisting diseases (Table 2 and Table 3). Additionally, the influence of each specific coexisting disease on every HRQoL dimension was thoroughly investigated. The findings from this detailed examination are presented in Table S2, as referenced at the end of Section 3.1. 

In the Interference with Life dimension, women were found to experience greater interference in life due to T1DM compared to men. Furthermore, among emerging adults, those aged between 25 and 29 years reported more significant disruption in their lives due to diabetes than their younger counterparts aged 18 to 24 years. Additionally, a diagnosis at or after the age of 18 was associated with more pronounced interference in life than in those diagnosed before this age. Interestingly, individuals who have been living with T1DM for less than five years reported greater life interference compared to those who have managed the condition for over five years. Similarly, the presence of a coexisting condition alongside T1DM was linked to increased interference in life, highlighting the compounded challenges faced by emerging adults managing multiple health conditions. 

With respect to the Well-Being dimension, men reported higher levels of well-being compared to women, with this disparity being of approximately medium effect size. Moreover, emerging adults with an A1C level lower than 53 mmol/mol exhibited greater well-being than those with an A1C of 53 mmol/mol or higher. The presence of a coexisting condition alongside T1DM was found to reduce well-being levels compared to individuals without additional health conditions, also characterized by a medium effect size. 

Similarly, in Concern about the Condition dimension, a marked difference was noted between sex, with women exhibiting greater concern than men. Furthermore, emerging adults with an A1C level of 53 mmol/mol or higher also showed increased concern about their condition compared to those with a level below 53 mmol/mol. The level of concern about diabetes was observed to be higher in individuals with a coexisting condition, as opposed to those with only T1DM. In addition, those not currently studying reflected higher concern about diabetes than those who were students at that moment. 

In the Self-Care dimension, individuals with an A1C level below 53 mmol/mol demonstrated significantly higher self-care scores compared to those with an A1C level of 53 mmol/mol or higher. This difference was characterized by a medium effect size.

### 3.3. Relationships of Participants’ Quality of Life with Their Demographic Characteristics and Clinical Diabetes Determinants

The ANOVA analysis demonstrated the association of factors such as the civil status, geographic dispersion, BMI, living situation, type of education, and employment status on each HRQoL in the T1DM dimension (Table S3).

A significant correlation was observed between Well-Being and BMI classifications. Notably, there were marked differences in well-being levels between emerging adults of normal weight and those classified as having class I obesity.

Similarly, another significant correlation was identified between Well-Being and education levels. Considerable differences in Well-Being were observed among emerging adults with varying levels of education. The lowest levels of Well-Being were found in individuals who had only completed compulsory secondary education, in contrast to those with higher educational attainments, including high school, higher degree, undergraduate, and postgraduate levels.

While the ANOVA analysis revealed significant correlations between civil status and living situation with aspects of Well-Being, the post hoc analysis did not confirm these differences. However, the ANOVA results suggested a higher level of well-being among single individuals and increased interference in life for those living independently.

**Figure 2:**
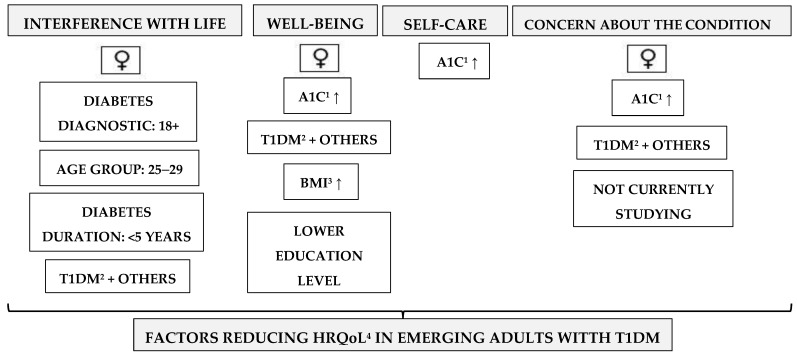
Key factors reducing Health-Related Quality of Life in Type 1 diabetes mellitus—insights from *t*-Student and ANOVA. ^1^ A1C: Glycosylated hemoglobin A1C. ^2^ T1DM: Type 1 diabetes mellitus. ^3^ BMI: Body Mass Index. ^4^ HRQoL: Health-Related to Quality of Life.↑: Increase in variable value.

To provide a comprehensive visual overview of the findings from both the *t*-Student and ANOVA tests, a detailed pictorial scheme is presented following this section (Figure 2). This summary visually encapsulates the key insights and patterns that emerged from the analyses conducted in Section 3.2 and Section 3.3.

### 3.4. Pearson Correlations between Sex, Age, Body Mass Index, A1C, Age at Diagnosis, Disease Evolution Time, and HRQOL in T1DM Dimensions of Vida1 Questionnaire

Pearson correlation analysis indicated that Interference with Life was related to sex, age, and age at diagnosis. In contrast, Self-Care was found correlated solely with A1C levels. Both Concern about the Condition and Well-Being demonstrated correlations with sex and A1C levels, with Well-Being also showing a relationship with BMI. The duration of diabetes did not show significant correlations with any of the HRQoL in T1DM dimensions. Detailed intercorrelations among other variables are presented in Table 4.

### 3.5. Multiple Regression Analyses for Each Dimension of Quality of Life in Type 1 Diabetes (Interference with Life, Self-Care, Well-Being, and Concern about the Condition)

In the multivariable analysis, only variables that were statistically significant in the univariate analysis were included. Age, with a predictive power of 2.8%, was found to predict Interference with Life. Self-Care was predicted by A1C, which accounted of 11.2% of the variance (R^2^). Well-Being was predicted by sex, A1C, and BMI, collectively contributing to an R^2^ of 11.5%. Concern about the Condition had two significant predictors: sex and A1C, with an R^2^ of 3.8%. Details of each regression model are in Table 5.

In this study, no correction for multiple testing—such as the Bonferroni method—was applied. However, it is noteworthy that even if such a correction had been employed, the significance of all predictors would have been retained, with *p*-values remaining below 0.0125 (calculated as α = 0.05/4). The only exception to this was A1C as a predictor of Concern about the Condition, which did not maintain its significance under this adjusted threshold.

## 4. Discussion

This study reveals that in Andalusia, emerging adults with T1DM generally experience good HRQoL in T1DM, characterized by high levels of well-being and self-care, coupled with low levels of life interference. However, concerns related to diabetes remain significant. 

Despite the apparent homogeneity, variations are observed within the Interference with Life dimension, particularly among women and those living independently. Factors such as coexisting conditions, diagnosis of T1DM at or after age 18, or a shorter duration of T1DM may contribute to increased interference with life. Age emerged as the key predictor in this dimension. Regarding Self-Care, participants with an A1C level below 53 mmol/mol exhibited higher self-care levels, with A1C identified as predictor. Lower well-being levels were associated with being female, having an A1C above 53 mmol/mol, comorbidities, marital status, obesity, or lower educational levels. Predictors for Well-Being included A1C, BMI, and sex. Concern about diabetes was notably higher among women, individuals with A1C above 53 mmol/mol, and those not currently engaged in studies. Sex and A1C were identified as predictive factors for this dimension.

When compared to findings in the study by Alvarado-Martel et al. [28] on emerging adults with T1DM, similar mean scores can be observed in the dimensions of Interference with Life (27.4 SD 10.3 vs. 27.8 SD 7.8) and Self-Care (40.2 SD 5.5 vs. 40.2 SD 7.7). However, greater differences emerge in the Well-Being (21.3 SD 5.1 vs. 23.1 SD 4.7) and Concern about the Condition (17.9 SD 4.9 vs. 18.5 SD 3.7) dimensions, with our participants showing lower levels of Well-being and Concern about the Condition.

In the study by Alvarado-Martel et al. [28], the levels of A1C <53 mmol/mol were found to correlate with higher Self-Care scores and a lower concern about diabetes, a finding that aligns with the results of the present study. Additionally, they reported an inversely proportional relationship between A1C levels and Well-Being. Similarly, the systematic review by Pérez-Fernández et al. [13] suggests that higher A1C levels are associated to a reduced cognitive well-being.

From childhood to emerging adulthood, there is often a gradual decline in glycemic target [1]. In this context, while the study by Kent and Quinn [32] on emerging adults with T1DM found no relationship between A1C levels and HRQoL in T1DM, our study, along with others involving children, adolescents, emerging adults, and adults [10,12,15,33,34], demonstrates a clear association. However, the longitudinal study by Stahl-Pehe et al. [16] with adolescents and emerging adults with T1DM did not establish causality in this correlation. Furthermore, in Moawd’s research [14], which included university students with both T1DM and type 2 diabetes, the relationship between A1C levels and both the physical and mental components of diabetes distress was found to be more pronounced in participants with T1DM.

The duration of diabetes has been identified as a predictive factor of HRQoL in T1DM [35]. Consistent with this, participants in our study who have had T1DM for less than five years reported greater interference with life. Contrarily, in the study by Alvarado-Martel et al. [28], an inverse correlation was found between the duration of diabetes and well-being. This relationship between the duration of the condition and HRQoL in T1DM has also been noted in research on emerging adults with T1DM by Moawd and Stahl-Pehe et al. [14,19]. Additionally, the age at diagnosis affects HRQoL in T1DM outcomes. In the present study, a diagnosis after the age of 18 was associated with a greater interference with life. Similarly, the study by Stahl-Pehe et al. [19], which compared two groups of adults under 31, one with an onset of disease in childhood and the other with onset in adulthood, found that the latter group experienced greater diabetes distress.

The present study identifies age as a predictor of HRQoL in T1DM, with emerging adults aged 25 to 29 years reporting a greater interference with life. This observation aligns with the findings by Vallis et al. [20], where participants aged 25 to 30 years with T1DM exhibited lower levels of HRQoL and heightened concern about diabetes compared to those aged 18 to 24 years. Such differences in the HRQoL in T1DM can be observed even within the early emerging adulthood bracket (ages 18 to 25) [6], but also when compared to younger age groups (ages 8–12 and 13–18) [10]. In contrast, the study by Kent and Quinn [32], which involved participants with T1DM aged 18 to 35 years, did not find evidence indicating that age or duration of diabetes had a significant impact on HRQoL in T1DM.

In our study, sex emerged as a predictor for the dimensions Well-Being and Concern about the Condition. This aligns with Alvarado-Martel et al. [28], who observed sex-based differences in these dimensions, with women showing higher levels of concern and lower well-being compared to men. Our findings also indicated that women had a higher score for the Interference with Life dimension than men. The trend of lower levels of HRQoL in T1DM and greater diabetes-related concern among women with respect to men has been widely discussed in the literature covering emerging adulthood and other age groups, regardless of the type of diabetes, blood glucose levels, or frequency of hyperglycemia episodes [6,13,15,17,22]. However, some studies have not found sex-based differences, yet highlight the importance of considering specific aspects related to sex in future research, such as the impact of menstruation [14,32,33].

With respect to the presence of comorbidities, overweight or obesity measured through the BMI emerged as predictor for Well-Being. This aligns with findings from Moawd’s study involving university students with diabetes [14]. Research on the impact of comorbid conditions alongside T1DM on HRQoL in T1DM is still limited [23,34]. The presence of diabetes-related complications has been shown to affect HRQoL in T1DM during emerging adulthood and other life stages [14,33]. Raymakers et al. [34] noted that mental health conditions in adults with T1DM were predictive of poorer HRQoL in T1DM. Similarly, Shapira et al.’s study [23] concerning adolescents with T1DM indicated that the presence of two or more comorbid conditions or a mental health condition was associated with a lower HRQoL in T1DM. In our study, coexisting conditions such as mental health issues or hypothyroidism, as well as those categorized as “others” were found to increase interference with life. While Shapira et al. [23] noted some adolescents displaying resilience in maintaining HRQoL in T1DM despite additional conditions, Kent and Quinn’s research [32] indicated that emerging adults have significant concerns about diabetes management and future complications. This is reflected in our study, where items like “I feel worried when I have high glycemia” and “I often worry about having future complications due to my diabetes” scored high. Bronner et al. [6] discovered that emerging adults without diabetes have better HRQoL compared to those having T1DM, yet those with T1DM scored better than peers with other chronic diseases with peers having other chronic diseases.

Other important milestones during emerging adulthood, such as educational achievements, living independently, and entering the job market, can impact HRQoL in T1DM. Alvarado-Martel et al. [28] found that individuals who completed secondary school and university education showed lower scores in the Concern about the Condition dimension than those who only completed primary education. In our study, this dimension was influenced when dividing the sample between those who were studying and those who were not, with the latter group showing greater concern levels. 

However, differences in Well-Being were more pronounced when considering levels of education. Participants living alone and independently declared greater interference with life. Accordingly, in the study on emerging adults with T1DM by Hanna et al. [36], lower levels of HRQoL in T1DM were also associated with living independently. In the present study, no significant differences in the HRQoL of patients with T1DM were observed in relation to employment status. This can be explained by the fact that in this stage, other questions are prioritized, such as the continuation of studies, as unemployment in adulthood is linked to lower HRQoL in T1DM [34]. Interestingly, single participants in our study show higher levels of well-being compared to their married counterparts. However, Willers et al. [37] found that being married in adults with T1DM was associated with lower levels of A1C. The complex interplay between romantic relationships and HRQoL in individuals with T1DM requires further investigation. 

The main strength of this study lies in the sample size, not only because it is considerably large, but also because it is representative of the region object of analysis. The sample size was calculated by taking into account the total number of emerging adults with type 1 diabetes in Andalusia, thereby ensuring the representativeness of our study population. Furthermore, there no data were lost as participants had to answer and complete every question in the questionnaire. An additional significant strength is the use of a validated questionnaire, originating from the same country of the study. This ensures cultural and linguistic accuracy of the responses, which is crucial for the validity of our findings.

Despite its strengths, this study has certain limitations. As a cross-sectional study, it is important to note that the causality of the associations found cannot be concluded. This highlights the need for future longitudinal studies to understand the evolution of HRQoL in T1DM throughout emerging adulthood. While economic status and ethnicity were not included in our study, potentially limiting the generalizability of our findings to other populations, we did incorporate variables such as civil status, educational level, and employment status. This approach allowed us to provide a broad perspective on other significant sociocultural factors. 

An inherent limitation of this study is the reliance on self-reported data, which introduces the potential for recall bias. This bias may have implications for both the direction and magnitude of our findings. Recall bias can lead to underreporting or overreporting of certain behaviors or health indicators, thus potentially skewing the associations observed. For instance, participants’ recollections of their A1C levels might not accurately reflect their actual medical records, potentially leading to discrepancies in reported versus actual diabetes management. This misreporting, whether intentional or unintentional, could affect the accuracy of the data on which our conclusions are based. While this limitation is a common challenge in survey-based research, efforts were made to minimize its impact by using validated questionnaires and providing clear instructions to participants. Nevertheless, the potential influence of recall bias on our results, especially in terms of A1C levels, between others parameters, must be acknowledged and considered when interpreting the findings. Future studies could benefit from incorporating objective measures, such as clinical records, to complement self-reported data and further validate the observed associations.

Additionally, a key limitation of our study is the methodology employed in analyzing comorbidities among emerging adults with T1DM. Our research uncovered a significant association between HRQoL dimensions and the presence of diverse comorbidities. These include mental health issues, hypothyroidism, lactose intolerance, cardiovascular diseases, skin problems, and gastroparesis, along with obesity. Additionally, less prevalent conditions like retinopathy and polycystic ovary syndrome were categorized as “others”. However, our analysis did not extend to investigating the cumulative effects of these comorbidities or their potential role as secondary to T1DM. The study’s findings bring to light the prevalence of these comorbidities in emerging adulthood, underscoring the imperative of early intervention to potentially avert future frailty and associated complications. This is particularly vital in light of the established correlation between blood glucose levels and the heightened risk of both physical and cognitive impairments, as evidenced in recent research on older adults with diabetes [38,39]. These revelations stress the importance of maintaining glycemic stability from a young age to reduce the likelihood of such comorbidities and their subsequent impact on HRQoL. Therefore, our study forms a foundational basis for subsequent research that delves into the complex relationship between T1DM and its comorbidities in younger cohorts, focusing specifically on the cumulative impact of multiple conditions on patient health and the advantages of holistic disease management strategies from the early stages of adulthood.

Finally, this study did not consider the use of diabetes devices despite the fact that the Andalusian Health Service provides the FreeStyle Libre 2 system for all people with T1DM, reflecting widespread access to this technology. Additionally, other continuous insulin infusion and glucose monitoring systems are also financed in certain cases. Considering the rapid technological advancements in diabetes care, future research should specifically examine the impact of different diabetes device models on HRQoL in T1DM.

Our findings have public health implications. Emerging adults with T1DM have a good HRQoL and a high level of self-care, suggesting that current diabetes management strategies are effective. However, the heightened level of concern about diabetes requires attention, given its potential to exacerbate mental health issues in a population that is already at a higher risk [40]. 

This study highlights several health-related factors, such a coexisting health conditions, diagnosis after age 18, and a shorter duration of T1DM, as potential disruptors to daily life. Maintaining an A1C level below 53 mmol/mol was identified as a key predictor of Self-Care. Although a higher percentage of emerging adults in Andalusia have an A1C below 53 mmol/mol compared to other study populations, this could be even further improved [9,10]. Nevertheless, it is crucial to extend the focus beyond just the A1C value to also consider glycemic variability and instability, time in range, and the frequency and severity of both hypoglycemias and hyperglycemias [41]. To achieve this, health practitioners, especially nurses, should implement regular health assessments and develop tailored public health interventions that address the specific needs of this population. 

## 5. Conclusions

In conclusion, emerging adults with T1DM in Andalusia generally demonstrate good HRQoL in T1DM, albeit with a high diabetes-related concern. Nonetheless, lower HRQoL in T1DM is more prevalent among women, those living independently, individuals with comorbidities, and those who are overweight or obese. Additionally, people diagnosed with T1DM during emerging adulthood or with a shorter duration of diabetes tend to have lower HRQoL. Sex, age, BMI, and A1C levels are identified as key predictors of HRQoL in T1DM.

## Figures and Tables

**Table 1:** Demographic characteristics of the participants (*n* = 362).

Variable	Mean	SD	n	%
**Sex (women)**			244	67.4
**Age (range: 18–29 years)**	22.8	3.4		
18–24			244	67.4
25–29			118	32.6
**Glycosylated hemoglobin**	55.2	12		
**(range: 26.8–79.2 mmol/mol)**				
<53 mmol/mol			172	47.1
≥53 mmol/mol			190	52.1
**Years of development of type 1 diabetes**	11.9	6.8		
**(range 1–28)**				
**Age at type 1 diabetes diagnosis**	10.9	6.7		
**(range 0–28)**				
**Body Mass Index (kg/m^2^)** ^1^	24.0	4.3		
(range: 17.15–48.68)				
Low weight			12	3.3
Normal weight			248	68.5
Pre-obesity or overweight			77	21.3
Obesity class 1			13	3.6
Obesity class 2			9	2.9
Obesity class 3			3	0.8
**Civil status**				
Single			295	81.5
Domestic partnership			62	17.1
Married			5	1.4
**Employment status**				
Does not work			178	49.2
Full-time work			86	23.8
Part-time work			37	10.2
Casual or temporary work			50	13.8
Self-employed			10	2.8
**Andalusia: geographic location**				
Almeria			22	6.1
Cadiz			38	10.5
Cordoba			32	8.8
Granada			53	14.6
Huelva			16	4.4
Jaen			21	5.8
Malaga			61	16.9
Seville			119	32.9

^1^ The WHO Classification of adults according to BMI.

**Table 2:** Differences between groups based on sociodemographic and clinical diabetes determinants using t-Student (Interference with Life and Self-Care).

		Interference with Life					Self-Care			
		Score Range: 12–60					Score Range: 5–25			
		n	Mean	SD	*t*	*d*	Mean	SD	*t*	*d*
Sex	Male	118	25.8	10.2	−2.009 *	0.23	40.7	5.5	1.169	0.13
	Female	244	28.1	10.3			40.0	5.5		
Age Group	18–24	244	26.3	10.0	−2.840 **	0.32	40.5	5.5	1.146	0.15
	25–29	118	29.6	10.4			39.7	5.5		
Glycosylated Hemoglobin	<53 mmol/mol	172	27.2	10.2	−0.297	0.03	41.9	4.4	5.722 ***	0.61
	≥53 mmol/mol	190	27.5	10.3			38.7	6.0		
Age at diagnosis	<18 years	294	26.6	10.1	−3.168 **	0.42	40.2	5.6	0.113	0.02
	≥18 years	68	30.9	10.5			40.2	5.2		
Diabetes evolution time	<5 years	62	30.3	11.4	2.450 *	0.33	40.9	5.2	1.132	0.15
	≥5 years	300	26.8	9.9			40.1	5.6		
Coexisting condition	No	157	25.3	9.1	−3.515 ***	0.37	40.6	5.5	1.291	0.13
	Yes	205	29.0	10.9			39.9	5.5		
Studying at that moment	No	100	28.1	11.0	0.853	0.10	40.1	5.7	−0.245	0.04
	Yes	262	27.1	10.0			40.3	5.5		

* *p* <0.05; ** *p* <0.01; *** *p* <0.001.

**Table 3:** Differences between groups based on sociodemographic and clinical diabetes determinants using t-Student (Well-Being and Concern about the Condition).

		Well-Being					Concern about the Condition			
		Score Range: 9–30					Score Range: 21–51			
		n	Mean	SD	*t*	*d*	Mean	SD	*t*	*d*
Sex	Male	118	23.2	5.0	5.188 ***	0.52	16.8	4.8	−3.198 **	0.36
	Female	244	20.4	4.9			18.5	4.7		
Age Group	18–24	244	21.5	5.1	0.986	0.11	17.7	4.7	−1.165	0.15
	25–29	118	20.9	5.0			18.4	4.9		
Glycosylated Hemoglobin	<53 mmol/mol	172	22.2	4.7	3.186 **	0.34	17.3	5.1	2.497 *	0.25
	≥53 mmol/mol	190	20.5	5.3			18.5	4.4		
Age at diagnosis	<18 years	294	21.2	5.2	−0.355	0.06	17.8	4.7	−1.459	0.21
	≥18 years	68	21.5	4.9			18.7	5.3		
Diabetes evolution time	<5 years	62	21.5	5.0	0.305	0.04	18.4	4.8	0.805	0.10
	≥5 years	300	21.3	5.1			17.9	4.8		
Coexisting condition	No	157	22.7	4.5	4.815 ***	0.51	17.4	4.7	−1.748	0.21
	Yes	205	20.2	5.3			18.3	4.8		
Studying at that moment	No	100	21.5	5.3	0.535	0.06	18.8	5.2	2.012 *	0.24
	Yes	262	21.2	5.0			17.6	4.6		

* *p* <0.05; ** *p* <0.01; *** *p* <0.001.

**Table 4:** Correlation results between dimensions of Health-Related Quality of Life and sociodemographic and clinical diabetes determinants.

Variables	Interference with Life	Self-Care	Well-Being	Concern about the Condition
Sex	0.0105 *		0.166*	−0.264 ***
Age	0.176 **			
Body Mass Index			−0.168 **	
Glycosylated hemoglobin		−0.335 ***	0.124 *	−0.182 ***
Age at diagnosis	0.116 *			

* *p* <0.05; ** *p* <0.01; *** *p* <0.001.

**Table 5:** Regression analysis examining sociodemographic and clinical diabetes determinants of each dimension of Health-Related Quality of Life.

	Interference with Life	
	Stand. C. Beta	Adjusted R^2^
Age	0.176	0.028
F (1.360) =11.563; *p* < 0.05; R^2^ = 0.031		
	**Self-Care**	
	Stand. C. beta	Adjusted R^2^
Glycosylated hemoglobin	−0.378 ***	0.112
F (3.358) =19.893; *p* < 0.05; R^2^ = 0.112		
	**Well-Being**	
	Stand. C. beta	Adjusted R^2^
Sex	−0.255 ***	0.067
Glycosylated hemoglobin	−0.173 ***	0.098
Body Mass Index	−0.142 **	0.115
F (3.358) = 16.672; *p* < 0.05; R^2^ = 0.123		
	**Concern about the Condition**	
	Stand. C. beta	Adjusted R^2^
Sex	0.166 **	0.025
Glycosylated hemoglobin	0.207 *	0.038
F (2.359) = 8.066; *p* < 0.05; R^2^ = 0.043		

* *p* <0.05; ** *p* <0.01; *** *p* <0.001.

## Data Availability

The data presented in this study are available upon reasonable request. The data are available from María-Ángeles Núñez-Baila (email: mnbaila@us.es).

## Supplementary Materials

The following supporting information can be downloaded at: https://www.mdpi.com/article/10.3390/jcm13010240/s1; Table S1: Descriptive analysis of the ViDa1 questionnaire in emerging adults with type 1 diabetes. Table S2: Differences between having or not having a coexisting condition other than type 1 diabetes using the *t*-Student. Table S3: Differences between groups based on sociodemographic and clinical diabetes determinants using ANOVA.

## References

[1] Khadilkar A., Oza C. (2022). Glycaemic control in youth and young adults: Challenges and solutions. Diabetes Metab. Syndr. Obes..

[2] Arnett J.J. (2020). Emerging adulthood: A theory of development from the late teens through the twenties. Am. Psychol..

[3] Arnett J.J. (2014). Emerging Adulthood: The Winding Road from the Late Teens through the Twenties.

[4] Lystad R.P., Pulido D.F., Peters L., Johnstone M., Ellis L.A., Braithwaite J., Wuthrich V., Amin J., Cameron C.M., Mitchell R.J. (2022). Feasibility of monitoring health and well-being in Emerging adults: Pilot longitudinal cohort study. JMIR Form. Res..

[5] Kata J. (2019). Quality of life in the youth perspective—systematization of definitions. Humanum.

[6] Bronner M.B., Peeters M.A.C., Sattoe J.N.T., Van Staa A.L. (2020). The impact of type 1 diabetes on young adults’ health-related quality of life. Health. Qual. Life Outcomes.

[7] Bekele B.T., Demie T.G., Worku F. (2022). Health-Related Quality-of-Life and associated factors among children and adolescents with type 1 diabetes mellitus: A Cross-Sectional Study. Pediatr. Health Med. Ther..

[8] Renard E., Ikegami H., Daher Vianna A.G., Pozzilli P., Brette S., Bosnyak Z., Lauand F., Peters A., Pilorget V., Jurišić-Eržen D. (2021). The SAGE study: Global observational analysis of glycaemic control, hypoglycaemia and diabetes management in T1DM. Diabetes Metab. Res. Rev..

[9] Beck R.W., Calhoun P., Kollman C. (2012). Use of Continuous Glucose Monitoring as an Outcome Measure in Clinical Trials. Diabetes Technol. Ther..

[10] Anderson B.J., Laffel L.M., Domenger C., Danne T., Phillip M., Mazza C., Hanas R., Waldron S., Beck R.W., Calvi-Gries F. (2017). Factors associated with diabetes-specific health-related quality of life in youth with type 1 diabetes: The Global TEENs study. Diabetes Care.

[11] Carels C., Wauters L., Outtier A., Baert F., Bossuyt P., Colard A., De Looze D., Ferrante M., Goegebuer A., Hauser B. (2021). Health literacy and quality of life in young adults from the Belgian Crohn’s disease registry compared to type 1 diabetes mellitus. Front. Pediatr..

[12] Svedbo Engström M., Leksell J., Johansson U.B., Borg S., Palaszewski B., Franzén S., Gudbjörnsdottir S., Eeg-Olofsson K. (2019). Health-related quality of life and glycaemic control among adults with type 1 and type 2 diabetes—A nationwide cross-sectional study. Health Qual. Life Outcomes.

[13] Pérez-Fernández A., Fernández-Berrocal P., Gutiérrez-Cobo M.J. (2023). The relationship between well-being and HbA1c in adults with type 1 diabetes: A systematic review. J. Diabetes.

[14] Moawd S.A. (2022). Quality of life in university students with diabetes distress: Type 1 and type 2 of diabetes differences. J. Diabetes Res..

[15] Naughton M.J., Yi-Frazier J.P., Morgan T.M., Seid M., Lawrence J.M., Klingensmith G.J., Waitzfelder B., Standiford D.A., Loots B. (2014). Longitudinal associations between sex, diabetes self-care, and health-related quality of life among youth with type 1 or type 2 diabetes mellitus. J. Pediatr..

[16] Stahl-Pehe A., Landwehr S., Lange K.S., Seid M., Lawrence J.M., Klingensmith G.J., Waitzfelder B., Standiford D.A., Loots B. (2017). Impact of quality of life (QoL) on glycemic control (HbA1c) among adolescents and emerging adults with long-duration type 1 diabetes: A prospective cohort-study. Pediatr. Diabetes.

[17] Castellano-Guerrero A.M., Guerrero R., Ruiz-Aranda D., Perea S., Pumar A., Relimpio F., Mangas M.A., Losada F., Martínez-Brocca M.A. (2020). Gender differences in quality of life in adults with long-standing type 1 diabetes mellitus. Diabetol. Metab. Syndr..

[18] Hanna K.M., Weaver M.T., Stump T.E., Guthrie D., Oruche U.M. (2014). Emerging adults with type 1 diabetes during the first year post-high school: Perceptions of parental behaviors. Emerg. Adulthood.

[19] Stahl-Pehe A., Bächle C., Bódis K., Zaharia O.P., Lange K., Holl R.W., Roden M., Rosenbauer J., Roden M., GDS Group (2023). Comparison of diabetes distress and depression screening results of emerging adults with type 1 diabetes onset at different ages: Findings from the German early-onset T1D study and the German Diabetes Study (GDS). Diabetol. Metab. Syndr..

[20] Vallis M., Willaing I., Holt R.I.G. (2018). Emerging adulthood and Type 1 diabetes: Insights from the DAWN2 Study. Diabet. Med..

[21] Ramchandani N., Way N., Melkus G.D., Sullivan-Bolyai S. (2019). Challenges to diabetes self-management in emerging adults with type 1 diabetes. Diabetes Educ..

[22] Dłużniak-Gołaska K., Szostak-Węgierek D., Panczyk M., Szypowska A., Sińska B. (2019). May gender influence the quality of life in children and adolescents with type 1 diabetes?. Patient Prefer. Adherence.

[23] Shapira A., Harrington K.R., Goethals E.R., Volkening L.K., Laffel L.M. (2021). Health-Related Quality of Life (HRQOL) in youth with type 1 diabetes: Associations with multiple comorbidities and mental health conditions. Diabet. Med..

[24] Babiker A., Al Aqeel B., Marie S., Omer H., Bahabri A., Al Shaikh A., Zahrani N., Badri M., Al Dubayee M., Al Alwan I. (2021). Quality of life and glycemic control in saudi children with type 1 diabetes at different developmental age groups. Clin. Med. Insights Endocrinol. Diabetes.

[25] Rostaminasab S., Nematollahi M., Jahani Y., Mehdipour-Rabori R. (2023). The effect of family-centered empowerment model on burden of care in parents and blood glucose level of children with type I diabetes family empowerment on burden of care and HbA1C. BMC Nurs..

[26] Palladino D.K., Helgeson V.S., Reynolds K.A., Becker D.J., Siminerio L.M., Escobar O. (2013). Emerging adults with type 1 diabetes: A comparison to peers without diabetes. J. Pediatr. Psychol..

[27] D’Amico R.P., Pian T.M., Buschur E.O. (2023). Transition from Pediatric to Adult Care for Individuals With Type 1 Diabetes: Opportunities and Challenges. Endocr. Pract..

[28] Alvarado-Martel D., Ruiz Fernández M.A., Vigaray M.C., Carrillo A., Boronat M., Montesdeoca A.E., Chávez L.N., Sánchez M.P., Quevedo P.L., Suárez A.D.S. (2017). ViDa1: The development and validation of a new questionnaire for measuring health-related quality of life in patients with type 1 diabetes. Front. Psychol..

[29] Kwak S.G., Kim J.H. (2017). Central limit theorem: The cornerstone of modern statistics. Korean J. Anesthesiol..

[30] Ernstsson O., Burström K., Heintz E., Mølsted Alvesson H. (2020). Reporting and valuing one’s own health: A think aloud study using EQ-5D-5L, EQ VAS and a time trade-off question among patients with a chronic condition. Health Qual. Life Outcomes.

[31] Mundfrom D.J., Perrett J., Schaffer J., Piccone A., Roozeboom M., Schumacker R.E., Beasley M.T. (2006). Bonferroni Adjustments in Tests for Regression Coefficients. Multiple Linear Regression View Points.

[32] Kent D.A., Quinn L. (2018). Factors that affect quality of life in young adults with type 1 diabetes. Diabetes Educ..

[33] Cho M.K., Kim M.Y. (2021). What affects quality of life for people with Type 1 diabetes?: A Cross-Sectional Observational Study. Int. J. Environ. Res. Public. Health.

[34] Raymakers A.J.N., Gillespie P., O’Hara M.C., Griffin M.D., Dinneen S.F., O’Hara M.C. (2018). Factors influencing health-related quality of life in patients with Type 1 diabetes. Health Qual. Life Outcomes.

[35] Jendle J., Ericsson Å., Hunt B., Valentine W.J., Pollock R.F. (2018). Achieving good glycemic control early after onset of diabetes: A cost-effectiveness analysis in patients with type 1 diabetes in Sweden. Diabetes Ther..

[36] Hanna K.M., Weaver M.T., Slaven J.E., Fortenberry J.D., Dimeglio L.A. (2014). Diabetes-Related quality of life and the demands and burdens of diabetes care among emerging adults with type 1 diabetes in the year after High School Graduation. Res. Nurs. Health.

[37] Willers C., Iderberg H., Axelsen M., Dahlström T., Julin B., Leksell J., Lindberg A., Lindgren P., Muth K.L., Svensson A.M. (2018). Sociodemographic determinants and health outcome variation in individuals with type 1 diabetes mellitus: A register-based study. PLoS ONE.

[38] Mone P., Gambardella J., Lombardi A., Pansini A., De Gennaro S., Leo A.L., Famiglietti M., Marro A., Morgante M., Frullone S. (2022). Correlation of physical and cognitive impairment in diabetic and hypertensive frail older adults. Cardiovasc. Diabetol..

[39] Mone P., De Gennaro S., Frullone S., Marro A., Santulli G. (2023). Hyperglycemia drives the transition from pre-frailty to frailty: The Monteforte study. Eur. J. Intern. Med..

[40] Benton M., Cleal B., Prina M., Baykoca J., Willaing I., Price H., Ismail K. (2023). Prevalence of mental disorders in people living with type 1 diabetes: A systematic literature review and meta-analysis. Gen. Hosp. Psychiatry.

[41] Elsayed N.A., Aleppo G., Aroda V.R., Bannuru R.R., Brown F.M., Bruemmer D., Collins B.S., Hilliard M.E., Isaacs D., Johnson E.L. (2023). 6. Glycemic Targets: Standards of Care in Diabetes—2023. Diabetes Care.

