# Supplementary material for: Sociodemographic and Clinical Determinants on Health-Related Quality of Life in Emerging Andalusian Adults with Type 1 Diabetes: A Cross-Sectional Study
Source: J Clin Med. 2023 Dec 31;13(1):240. doi: 10.3390/jcm13010240 (PMC10779847; doi:10.3390/jcm13010240)
Supplement: Supplementary file 1 [file jcm-13-00240-s001.zip › jcm-2776356-supplementary.pdf]

Table S1. Descriptive analysis of the ViDa1 questionnaire in emerging adults with type 1 diabetes

	Items	Mean	SD
**INTERFERENCE WITH LIFE**			
1	Having diabetes is a problem for my social relationships (i.e., with friends. work colleagues. partner. etc.).	1.9	1.2
2	I feel different because of my diabetes.	2.5	1.3
3	Having to inject insulin is a daily problem for me.	2.1	1.2
4	Having diabetes limits my social life and free time activities (eating out. celebrations. trips. etc.).	2.4	1.4
5	My life has been changed by having diabetes	3.6	1.4
6	Having diabetes makes my relationship with my family more difficult.	1.7	1.1
7	I feel limited professionally by my diabetes.	2.4	1.4
8	In spite of my diabetes, I can lead a normal life.	2.3	1.4
9	One or more complications of my diabetes worsen my quality of life because it limits/they limit me physically.	3	1.5
10	Everyday life with diabetes represents an added source of stress.	1.5	1.1
11	I worry that other people know that I have diabetes.	1.7	1.1
12	My sex life is limited by my diabetes.	2.2	1.5
**Total score (range scale of score: 12-60)**		**27.4**	**10.3**
**SELF-CARE**			
13	I am happy with my involvement in the everyday self-care of my diabetes.	2.3	1.1
14	The level of training/knowledge I have about my diabetes helps me to maintain good control over it.	4.1	1.0
15	The training I have in carbohydrate counting allows flexibility in my diet.	3.9	1.1
16	I am happy with the way I cope with my diabetes.	3.6	1.1
17	I am motivated to take part in the care of my diabetes.	3.7	1.1
18	I adjust the insulin dose to my diet to obtain good control.	4.5	0.8
19	I am satisfied with my pharmacological treatment because it helps me to control my diabetes.	4.2	1.0
20	At this moment, I am satisfied with my glycemic control (glycosylated hemoglobin).	3.4	1.3
21	The management of my diabetes is part of my normal everyday life.	4.3	1.0
22	I consider that I have flexibility and freedom in my diet despite having diabetes.	4.1	1.1
23	I find it hard to carry out the daily controls (glycaemia).	2.1	1.3
**Total score (range of score:11-55)**		**40.2**	**5.5**
**WELL-BEING**			
24	I get plenty of rest and I sleep well at night.	3.3	1.3
25	I feel physically fine.	3.7	1.1
26	I feel psychologically fine.	3.3	1.3
27	I have other illnesses as a result of my diabetes which have a negative effect on my quality of life.	4.2	1.3
28	I am satisfied with the time I spend doing physical activity.	3.0	1.4
29	I think that in general my quality of life is good.	3.8	1.0
**Total score (range of score: 6-30)**		**21.3**	**5.1**
**CONCERN ABOUT THE CONDITION**			
30	I am frightened of having hypoglycemia (sugar level drop).	3.3	1.4
31	I often worry about having a hypoglycemia.	3.3	1.4
32	I feel worried when I have high glycemia.	4.1	1.0
33	I often worry about having complications in the future due to my diabetes.	4.1	1.1
34	I often worry about being admitted to hospital because I cannot control my diabetes.	3.1	1.6
**Total score (range of score: 5-25)**		**17.9**	**4.9**

Table S2. Differences between having or not having a coexisting condition other than type 1 diabetes using the t-Student.

			INTERFERENCE WITH LIFE Score range: 12-60				SELF-CARE Score range: 5-25				WELL-BEING Score range: 9-30				CONCERN ABOUT THE CONDITION Score range: 21-51			
		N	Mean	SD	*t*	*d*	Mean	SD	*t*	*d*	Mean	SD	*t*	*d*	Mean	SD	*t*	*d*
MENTAL HEALTH CONDITION	No	357	27.5	10.2	-2.118*	0.93	40.2	4.5	-0.401	0.02	21.4	5.1	2.257*	1.12	18.0	4.8	0.160	0.07
	Yes	5	37.0	10.2			41.2	4.2			16.2	4.1			17.6	6.4		
CELIAC DISEASE	No	335	27.4	10.4	0.260	0.05	40.2	5.6	-0.079	0.02	21.3	5.1	0.696	0.14	17.9	4.9	-0.273	0.07
	Yes	27	26.9	8.7			40.3	5.3			20.6	4.9			18.2	4.2		
LACTOSE INTOLERANCE	No	345	27.2	10.2	-1.367	0.31	40.3	5.4	1.471	0.31	21.4	5.0	2.048*	0.14	18.0	4.8	1.140	0.25
	Yes	7	30.7	12.3			38.3	7.3			18.8	5.8			16.7	5.4		
ASTHMA	No	332	27.3	10.2	-0.584	0.11	40.3	5.5	1.017	0.20	21.4	5.1	-1.784	0.48	17.9	4.8	-0.386	0.24
	Yes	30	28.4	11.3			39.2	5.7			19.7	5.5			18.3	4.6		
CARDIOVASCULAR DISEASE	No	352	27.3	10.2	-1.317	0.39	40.3	5.9	0.647	0.22	21.4	5.0	2.589**	0.75	17.9	4.8	-0.172	0.09
	Yes	10	31.6	11.8			39.1	5.0			17.2	6.2			18.2	4.9		
SKIN PROBLEMS	No	301	27.0	10.1	-1.583	0.22	40.3	5.6	0.842	0.11	21.7	5.0	3.219***	0.45	17.9	4.8	-0.513	0.06
	Yes	61	29.3	10.9			39.7	5.2			19.4	5.3			18.2	4.9		
FOOD ALLERGIES	No	346	27.2	10.1	-1.719	0.19	40.2	5.6	-0.188	0.04	21.3	5.8	0.831	0.21	18.0	4.8	0.110	0.04
	Yes	16	31.7	12.4			40.4	5.1			20.3	4.8			17.8	5.3		
NON FOOD ALLERGIES	No	288	27.5	10.2	0.322	0.05	40.2	5.6	0.022	0.00	21.3	5.1	0.338	0.04	18.0	4.8	0.399	0.06
	Yes	74	27.0	10.7			40.2	5.2			21.1	5.3			17.7	5.0		
GASTROPARESIS	No	341	27.1	10.0	-1.877	0.48	40.2	5.6	-0.377	0.09	21.5	5.0	3.124**	0.66	17.9	4.7	0.243	0.05
	Yes	20	32.8	13.4			40.7	5.1			17.9	5.9			17.6	6.2		
HYPOTHYROIDISM	No	326	27.0	10.2	-2.238*	0.39	40.2	5.6	-0.357	0.06	21.4	5.2	1.148	0.22	17.9	4.9	-0.808	0.06
	Yes	36	31.0	10.5			40.5	5.0			20.4	4.3			18.6	4.2		
OBESITY	No	338	27.3	10.3	-0.415	0.01	40.3	5.4	0.731	0.15	21.5	5.1	3.161**	0.69	17.9	4.8	-0.935	0.22
	Yes	23	28.2	9.8			39.4	6.4			18.1	4.8			18.9	4.5		
OTHER†	No	324	26.7	10.0	-3.851***	0.63	40.4	5.5	1.718	0.28	21.7	4.9	4.993***	0.82	17.8	4.8	-1.223	0.21
	Yes	38	33.3	11.1			38.8	5.9			17.5	5.4			18.8	4.6		

* $p < 0.05$; ** $p < 0.01$; *** $p < 0.001$

†Other: anemia, candidiasis, constipation, fibromyalgia, human papillomavirus, hypercholesterolemia, hyperthyroidism, irritable bowel syndrome, irritable colon, migraines, polycystic ovary syndrome, proteinuria, renal failure, retinopathy, tendinitis, varicose veins.

Table S3. Differences between groups based on sociodemographic and clinical diabetes determinants using ANOVA.

		**INTERFERENCE WITH LIFE**				**SELF-CARE**				**WELL-BEING**				**CONCERN ABOUT THE CONDITION**			
		**Score range: 12-60**				**Score range: 5-25**				**Score range: 9-30**				**Score range: 21-51**			
	GROUPS	Mean	SD	*F*	η^2	Mean	SD	*F*	η^2	Mean	SD	*F*	η^2	Mean	SD	*F*	η^2
CIVIL STATUS	Single	27.3	10.2			40.3	5.3			21.6	4.9			17.7	10.2		
	Domestic partnership	27.4	11.1	0.653	0.004	40.1	6.5	0.227	0.001	20.2	5.9	3.193*	0.016	19.1	11.1	2.754	0.014
	Married	32.6	3.4			38.6	6.2			17.6	5.9			19.6	3.4		
GEOGRAPHIC LOCATION	Almeria	26.4	7.9			39.5	6.9			21.1	4.4			18.6	4.0		
	Cadiz	27.3	10.2			39.2	6.9			20.3	5.6			19.6	5.1		
	Cordoba	25.3	7.2			41.6	5.9			22.4	4.4			16.2	5.2		
	Granada	28.1	10.7	1.883	0.036	40.2	5.1	1.875	0.036	20.6	5.1	1.823	0.035	18.5	4.8	1.642	0.031
	Huelva	32.3	13.1			38.5	4.2			19.7	6.1			18.4	4.1		
	Jaen	29.0	11.5			41.1	6.8			21.8	6.4			18.2	4.3		
	Malaga	29.9	11.5			38.8	6.1			20.2	4.9			17.8	5.0		
	Seville	25.6	9.7			41.1	4.9			22.3	4.8			17.5	4.7		
BODY MASS INDEX	Low weight	30.8	10.5			39.5	6.3			19.4	4.1			19.8	4.2		
	Normal weight	27.2	10.6			40.3	5.4			21.9	5.0			17.5	4.8		
	Pre-obesity or overweight	27.2	9.9	0.465	0.007	40.8	5.3	1.482	0.021	20.7	5.2	4.142**	0.06	18.9	4.7	1.757	0.055
	Class I obesity	27.3	7.3			38.0	6.9			17.5	4.9			18.4	4.8		

	Class II obesity	30.2	9.3			36.4	5.6			17.2	3.9			19.9	3.8		
	Class III obesity	29.7	11.5			40.3	6.4			19.3	5.8			16.3	5.0		
LIVING SITUATION	With parents or legal guardians	26.9	10.2			40.8	5.3			21.6	5.0			17.7	4.6		
	With partner	29.6	11.0			38.7	6.5			20.2	5.8			18.5	5.6		
	Alone/Independent	32.5	11.9			39.5	4.2			22.0	4.7			20.2	3.9		
	With other relatives	20.5	7.8	2.459*	0.041	43.5	0.7	1.602	0.026	21.5	10.6	0.848	0.014	22.0	4.2	1.573	0.026
	With roommates	25.4	8.3			39.5	5.6			20.9	4.6			17.4	4.6		
	University residence hall	19.8	4.4			40.5	5.3			20.2	2.9			15.2	6.2		
	Other	33.0	8.5			44.0	5.7			19.0	5.7			17.5	3.5		
FINISHED EDUCATION	Primary education	31.8	11.9			41.8	1.7			19.5	4.8			21.0	1.8		
	Compulsory secondary-school education	29.3	12.5			38.8	6.4			17.9	6.0			19.6	5.3		
	High school	26.0	9.9			41.5	6.2			21.8	5.1			17.0	4.8		
	Vocational Intermediate vocational training	28.1	1.4	2.988	0.015	39.7	6.8	1.034	0.020	20.6	6.0	2.988**	0.056	19.1	3.9	1.554	0.031
	Higher vocational training	26.0	10.5			39.6	5.5			22.2	5.1			17.8	4.9		
	University degree	28.0	9.7			40.2	4.8			21.3	4.7			17.9	4.6		
	Master or postgraduate	27.5	9.9			40.6	5.0			21.8	4.2			17.7	5.2		
	PhD studies	21.3	7.8			37.7	10.2			26.7	4.2			14.0	6.2		

EMPLOYMENT STATUS	Does not work	30.8	10.5			40.6	5.4			21.2	5.1			18.0	4.4		
	Full-time work	27.2	10.6			40.4	6.1			21.8	5.4			17.8	5.6		
	Part-time work	27.2	9.9	2.146	0.024	38.4	5.9	1.935	0.021	20.8	5.1	1.733	0.019	18.8	4.4	1.41	0.016
	Casual or temporary work	27.3	7.3			39.7	4.6			20.4	4.6			18.1	4.9		
	Self-employment work	30.2	9.3			42.9	3.2			24.6	5.1			14.8	4.7		

* $p < 0.05$; ** $p < 0.01$; *** $p < 0.001$
